# Memory Self-Efficacy Beliefs Modulate Brain Activity when Encoding Real-World Future Intentions

**DOI:** 10.1371/journal.pone.0073850

**Published:** 2013-09-03

**Authors:** Grégoria Kalpouzos, Johan Eriksson

**Affiliations:** 1 Umeå Center for Functional Brain Imaging (UFBI), Umeå, Sweden; 2 Department of Integrative Medical Biology, Physiology Section, Umeå University, Umeå, Sweden; 3 Aging Research Center (ARC), Karolinska Institute and Stockholm University, Stockholm, Sweden; University of Surrey, United Kingdom

## Abstract

**Background:**

While the use of different cognitive strategies when encoding episodic memory information has been extensively investigated, modulation of brain activity by memory self-efficacy beliefs has not been studied yet.

**Methodology/Principal Findings:**

Sixteen young adults completed the prospective and retrospective metamemory questionnaire, providing individual subjective judgments of everyday memory function. The day after, using functional magnetic resonance imaging, the participants had to memorize real-world intentions (e.g., return a book to the library), which were performed later on in a virtual environment. Participants also performed offline cognitive tasks evaluating executive functions, working memory, and attention. During encoding, activity was found in medial temporal lobe, left prefrontal cortex, medial parietal regions, occipital areas, and regions involved in (pre)motor processes. Based on results from the questionnaire, the group was split into low and high memory self-efficacy believers. Comparison of encoding-related brain activity between the 2 groups revealed that the low memory self-efficacy believers activated more the hippocampus bilaterally, right posterior parahippocampal cortex, precuneus, and left lateral temporal cortex. By contrast, more activity was found in dorsal anterior cingulate gyrus for the high-memory believers. In addition, the low-memory believers performed more poorly at feature binding and (at trend) manipulating visuospatial information in working memory.

**Conclusion/Significance:**

Overall, these findings indicate that memory self-efficacy beliefs modulate brain activity during intentional encoding. Low memory self-efficacy believers activated more brain areas involved in visuospatial operations such as the hippocampus. Possibly, this increase reflects attempts to compensate for poor performance of certain neurocognitive processes, such as feature binding. By contrast, high-memory believers seemed to rely more on executive-like processes involved in cognitive control.

## Introduction

The neural correlates of episodic memory have been extensively studied using various neuroimaging techniques [1], [2], [3]. While most of the studies in the field were concerned with the remembering of past events, the definition of episodic memory also stipulates that this system enables self-projection into the future [4]. The interest for this human capability is quite recent, but sparked off several studies in which the participants had to project themselves in the future by imagining scenarios, given a cue-word [5], [6], [7]. Memory for the future has also been studied in the context of prospective memory, defined as the retrieval and execution of delayed intentions [8]. Most of the neuroimaging studies on prospective memory lack ecological validity, and few experiments have investigated the brain correlates related to the encoding of future intentions [9], [10].

Atance and O'Neill [11] connected the episodic future-thinking concept to prospective memory. Importantly, the authors emphasized the fact that *“(developing a plan) has received little attention in the prospective memory literature, yet could be the component most intimately linked with episodic future thinking*” (p. 533). To bridge the gap between future thinking and prospective memory, we developed a protocol in which participants were scanned while intentionally encoding specific real-world tasks (i.e., various errands to perform at familiar places), and subsequently performing them in a virtual environment. Findings related to prospective retrieval have been published previously [12]; here we focused on the encoding phase.

When individuals have to memorize future intentions, a factor that is likely to drive diverse encoding strategies is the knowledge or beliefs that each person has about his or her memory proficiency, i.e. self-efficacy [13], [14]. The self-efficacy concept stems from the self-perception theory, postulating that “Individuals come to *know* their own attitudes, emotions, and other internal states partially by inferring them from observations of their own overt behavior and/or the circumstances in which this behavior occurs” (see [15], p. 2). Indeed, although experimental studies demonstrated that memory performance has a strong influence on memory self-efficacy beliefs, the relationship is bidirectional, such that in turn, beliefs influence performance via strategy selection [16]. Whereas numerous neuroimaging studies have investigated either the effect of forced or unforced strategies on the episodic encoding and retrieval neural networks [17], or modulation of brain activity at retrieval according to subjects’ judgments about the specific task to come (prediction) or their confidence rating after task accomplishment [18], [19], a hypothetical modulation of brain activity at encoding according to individuals’ general memory self-efficacy beliefs (as opposed to specific self-efficacy beliefs, i.e. intimately connected to the task of interest, see [20]) has rarely, if ever, been tested.

In order to study whether general memory self-efficacy beliefs affect the pattern of activated brain areas during intentional encoding, we used a non-specific and validated questionnaire, the PRMQ (Prospective and Retrospective Memory Questionnaire, [21]), which was completed by the participants prior to the task. By using the global score as a subjective measure of memory abilities in everyday life, we aimed at exploring whether low or high memory self-efficacy beliefs (i.e., reporting more or less everyday memory difficulties) modulate the pattern of brain activity when encoding delayed real-life intentions (e.g., return a book to the library). Typically, the expectations would be that individuals with higher memory self-efficacy beliefs apprehend memory tasks with higher confidence and better strategies in comparison to those with lower self-efficacy beliefs [22]. Alternatively, considering the circumstances in which the memory task occurred (participants enrolled in a research protocol and performed the task in a scanner), one can expect not only an overall high motivation in all participants to do the task well, but perhaps an even higher motivation in those who reported lower memory self-efficacy in everyday life situations. We then hypothesized that participants with lower memory self-efficacy beliefs would either enhance some neurocognitive mechanisms such as attention, or use different cognitive processes to compensate for their own shortcomings to efficiently achieve the task. This hypothesis may also reflect real-life differences, where people, according to their beliefs in memory self-efficacy, adopt different behaviors and strategies to complete prospective tasks (for instance low-memory believers may use more mental visualization or written notes than high-memory believers, see [23]).

The relationships between subjective and objective memory measures remain unclear, especially among younger adults [24], [25]. Associations were usually found between subjective measures and objective performance, but overall the effect size is small [25], [26], [27]. Although a few studies showed significant associations between the PRMQ and performance obtained at experimental prospective memory tasks [28], [29], in general the associations are very weak (for review see [30]). One factor for the lack of strong associations could be unmatched evoked situations between the content of the metamemory questionnaire and the content of experimental tasks [25]. This is especially true regarding prospective memory, which is most often evaluated with laboratory tasks whose validity against naturalistic prospective memory situations remains debatable [30]. Even though memory self-efficacy beliefs seem not to predict so well prospective memory performance, such beliefs are built on the perception of our memory success and failure in everyday life. Since prospective memory is a multidimensional construct that involves several cognitive functions [23], [31], we further sought for relationships between PRMQ scores and objective measures such as executive functions, attention, and working memory. Differences in cognitive performances may explain differences in brain activity when encoding real-world prospective memory tasks according to self-efficacy beliefs, and reveal different encoding-related strategies.

## Methods

### Ethics Statement

The study was approved by the ethics committee of Umeå University. All participants gave written informed consent to participate.

### Participants

Sixteen healthy adults (mean age = 26.5±6.7 years old, range: 19–48, 6 females), inhabitants of the town of Umeå, Sweden, participated in this study. All were right-handed except one, and had normal or corrected-to-normal visual acuity. None of them reported a history of neurological or psychiatric illness, or current use of psychoactive drugs.

### Tasks

#### PRMQ questionnaire

**Figure 1:**
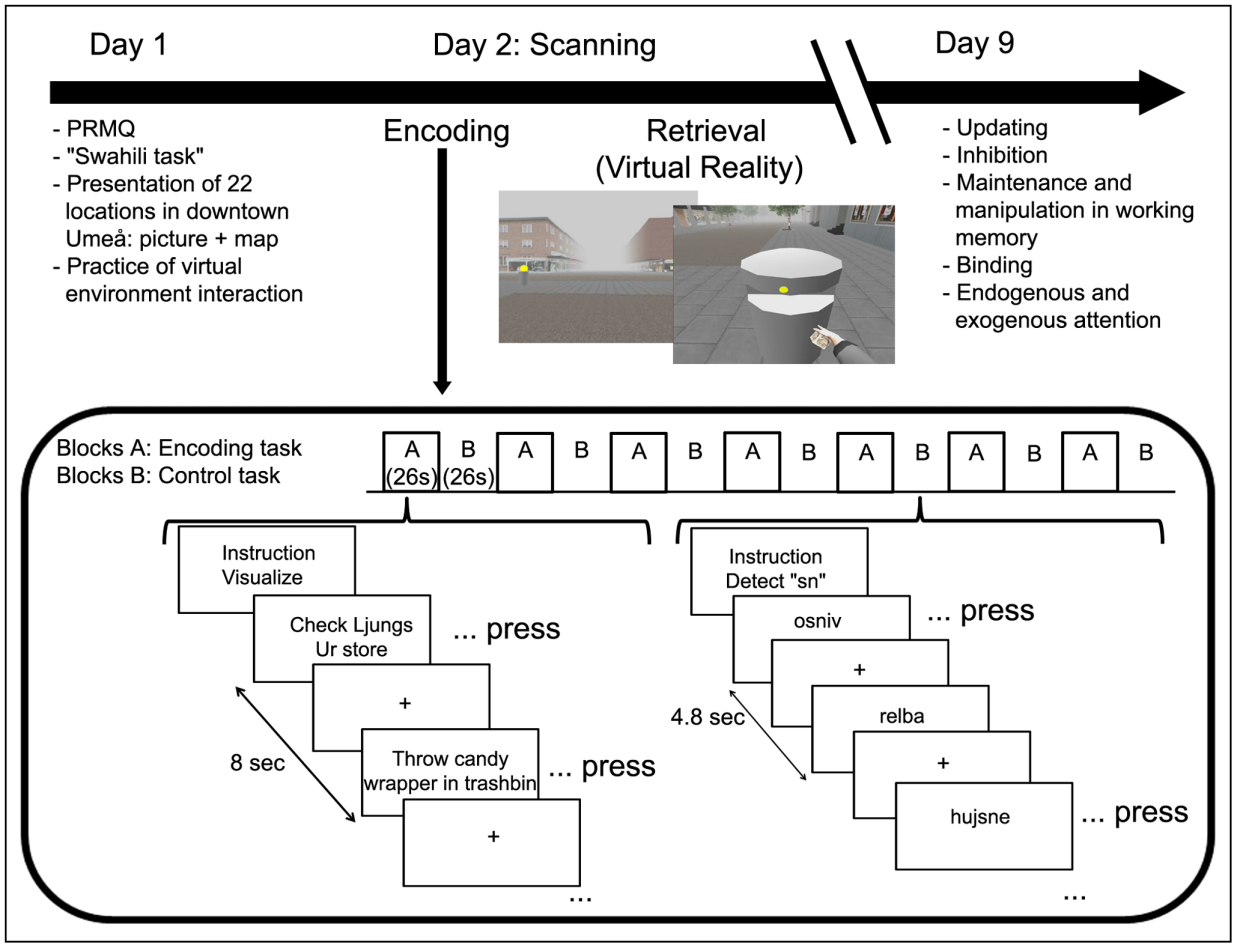
Study design.

The study design is depicted in Figure 1. On day 1, the participants were presented with the PRMQ questionnaire [21] in order to evaluate their subjective rating of memory function in everyday life. We decided to give the PRMQ prior to the cognitive tasks in order to avoid any influence of performance at the tasks of this study on the PRMQ ratings if the questionnaire was administered after. Our aim was to have “pure” self-efficacy beliefs, based on participants’ experience in everyday life. Briefly, this questionnaire contains 16 questions regarding retrospective (n = 8 questions) and prospective remembering (n = 8), short-term (n = 8) and long-term memory (n = 8), and self-cued (n = 8) and environmental cued based memory (n = 8). Thus, each question has multiple properties. An example of prospective memory question is: “Do you forget to buy something you planned to buy, like a birthday card, even when you see the shop?” The participants had to answer on a 5-point scale, from “never” (1 point) to “very often” (5 points). The range for the total score is 16–80 points. In the present study, we used the total score rather than the prospective-memory subscale, because it has been previously shown that retrospective aspects of episodic memory are used during construction and elaboration of future events [5]. Moreover, the prospective and retrospective memory subscales are highly correlated [30].

#### Familiarization with the material of the prospective memory task

On day 1, the participants were familiarized with the places they were going to interact with the day after (day 2) in the Magnetic Resonance Imaging (MRI) device for the encoding/retrieval prospective memory task. A picture of each place (taken from the virtual environment) was shown, together with a map of the town indicating its exact location. The aim of this phase was to ensure participants’ knowledge of all the places of Umeå relevant for the prospective memory task. The instructions were to look carefully at the places because they would have to interact with them the day after in the virtual environment. No memory instruction was given during this familiarization phase, and no mention was made that the future task was a memory task, but rather a navigation task. The participants also practiced for a few minutes on navigating in the virtual environment in order to evaluate their navigation skills and possible feeling of motion sickness. On that day, participants also performed a learning memory task, which was unrelated to the prospective memory task presented in this study (the “Swahili task”, see below).

#### Encoding intentions task

The encoding part of the prospective memory task took place on day 2 in the MRI scanner. It consisted of the presentation of sentences describing real-world-based tasks to be performed later in the virtual environment of downtown Umeå, presented one by one. The participants were instructed to read the sentence, press a button and picturing themselves performing the task (e.g., mail a letter, check out movie schedules at the movie theater, throw a coin in the fountain and make a wish, throw a candy wrapper in a trash bin). One block of 4 and 6 blocks of 3 sentences were displayed, interleaved with 7 blocks of a control task. The total number of to-be-encoded tasks was 22. The control task consisted of a low-level cognitive task, where participants had to detect the presence of 2 consecutive letters (“sn”) in pseudo-words, and they were instructed to press a button when “sn” was present. This task was chosen to control for the reading process and motor response (see [32]). At the beginning of each block, instructions appeared for two seconds (“Visualize” or “Detect sn”). Participants were given 8 seconds to read the task, press the button and imagine themselves performing it, while a fixation cross was displayed on the screen. The three-sentence blocks lasted for 26 seconds, and the four-sentence block lasted for 34 seconds. In the control task, the pseudo-words were displayed for 3.8 seconds each followed by a fixation cross for one second. For one block the subject had to detect the string 4 times and for the other blocks, 3 times. The visualization instruction for the encoding task was used in order to limit the use of very different cognitive strategies between subjects, and therefore narrow the investigation to the effect of subjective memory only. However, the use of additional strategies (e.g., verbal) cannot be completely excluded.

#### Offline cognitive tasks

One week after scanning, on day 9, the subjects returned and completed a test battery evaluating several cognitive functions. Within the executive domain, inhibition using the Stroop task [33], and updating using the letter memory task [34] were tested. Within working memory were measured visuospatial binding using the feature binding task [31], [35], as well as maintenance and manipulation of visuospatial information using the forward and backward Corsi cubes task [36]. Endogenous (top-down) and exogenous (bottom-up) attention was evaluated using Posner’s paradigm [37]. All tasks were computerized. The detail of these tasks can be found in Text S1. Learning slopes derived from a learning associative memory task (the “Swahili task”) were also used. The learning phase took place on the first day; the participants had to learn pairs of words, one word was in Swahili and the other was its translation in Swedish. At retrieval the Swahili words were displayed and the participants had to orally provide their translation in Swedish. This task consisted in repeated learning-recall loops and we here used the learning slopes over 4 learning-recall loops (this task also contained an fMRI phase that took place before the prospective memory task on day 2, see [38]).

Prospective memory performance in the scanner during the virtual reality task could not be used as a measure of memory due to experimentally induced ceiling effects: indeed, during the event-related prospective-memory virtual-reality fMRI retrieval task, before each of the 5 routes, the 4 to 5 errands were displayed again to the participants in order to avoid forgetting and other confusing situations such as unwanted deviation from the route of interest. Therefore, all participants performed well.

### Neuroimaging Procedure

The current study was carried out on a Philips 3.0 tesla using an 8 channel SENSE head coil (Philips Medical Systems, The Netherlands). For the functional high-speed echo-planar imaging (EPI) scanning, the following parameters were used: repetition time (TR) = 1512 ms for three participants and 1500 ms for the remaining participants (31 axial slices acquired), echo time (TE) = 30 ms, flip angle = 70°, field of view (FOV) = 22 cm×22 cm, matrix = 64×64, slice thickness = 4.65 mm, in-plane resolution = 3.44 mm×3.44 mm. To avoid signals arising from progressive saturation, ten dummy scans were performed prior to image acquisition. A high resolution T1-weighted MRI was also acquired for each subject using a 3D turbo field-echo sequence. Acquisitions consisted of a set of 170 adjacent sagittal slices, with a slice thickness of 1 mm, in-plane resolution = 0.3 mm×0.3 mm, and the following parameters: TR = 10.5 ms, TE = 5 ms, flip angle = 8°, FOV = 24 cm×24 cm, and matrix = 336×332 reconstructed to 800×800.

### Statistical Analyses

#### Behavior

The group of 16 participants was split into 2 groups of 8 according to their total PRMQ score, one group where memory self-efficacy was judged relatively low (“low memory self-efficacy believers”, scoring above the median [median = 34.5]), and the other group where memory self-efficacy was judged relatively high (“high memory self-efficacy believers”, scoring below the median).

Comparisons were performed between the 2 groups on the scores obtained at the cognitive tasks described above. We used analyses of covariance controlling for sex, because a trend toward a sex effect was found when splitting the group according to the PRMQ score (Table 1). In addition to the fact that sex was not a significant factor explaining PRMQ differences in the article reporting the norms [21], we were not interested in studying sex effects in the present study, and interaction involving this factor was excluded due to the small sample size.

**Table 1:** Behavioral data and comparisons between the low and high memory self-efficacy believers.

	All	Low Believers	High Believers	Comparisons*
Age	26.5 (6.7)	27 (8.8)	26 (4.3)	
Sex	6F–10M	5F–3M	1F–7M	Fisher’s exact p = .06 (1-tailed)/p = .12 (2-tailed)
PRMQ	34.7 (8.4)	41 (6)	28.4 (4.8)	F (1,13) = 14.85, p = .002
Associative Learning	18.8 (5.5)	20.8 (4.4)	16.8 (5.9)	F = 2.99, p = .11
VS Maintenance in WM	6.1 (1.6)	6 (1.5)	6.1 (1.8)	F = 0.07, p = .80
VS Manipulation in WM	5.9 (2)	4.9 (1.6)	6.9 (1.9)	F = 3.33, p = .09
Feature Binding in WM	29.1 (4.8)	27.1 (4.7)	31.1 (4.3)	F = 9.62, p = .008
Updating	2.2 (1.9)	2.1 (1.9)	2.3 (2)	F = 0.99, p = .34
Inhibition	197.7 (89.2)	186.7 (65)	208.7 (112)	F = 0.28, p = .61
Endogenous Attention	50 (22.7)	40.6 (18.9)	59.4 (23.4)	F = 1.16, p = .30
Exogenous Attention	66.7 (22.8)	62.1 (12.6)	71.3 (30.2)	F = 0.14, p = .72

Mean (Standard Deviation).

* Analyses of covariance, controlling for sex. Performance in inhibition and attention is expressed in ms (Inhibition = incongruent trials – congruent trials; Attention = invalid trials – valid trials).

Abbreviations: F = female; M = male; PRMQ = Prospective and Retrospective Memory Questionnaire; VS = visuospatial; WM = working memory.

#### Neuroimaging

Functional images were pre-processed and analyzed in SPM8 (Statistical Parametric Mapping, Wellcome Department of Imaging Science, Functional Imaging Laboratory, http://www.fil.ion.ucl.ac.uk/fil.html) implemented in Matlab 7.12.0 (Mathworks Inc, MA, US). After correcting for differences in slice timing within each image volume, all images were realigned to the first image volume acquired, then normalized to standard anatomic space defined by the Montreal Neurological Institute (MNI) atlas, and finally spatially smoothed using a full-width at half-maximum Gaussian kernel of 8×8×8 mm. T1-weighted images were also preprocessed in SPM8 using the default parameters of the “new segment” tool; the normalized gray matter images of the 16 participants were averaged in order to generate a binary gray matter mask that was used as an “explicit mask” for the group-level functional MRI analyses.

Both the encoding and control conditions were modeled as a fixed response (box-car) waveform convolved with the hemodynamic response function. Covariates of no interest included the six realignment parameters to account for motion artifacts. Single-subject statistical contrasts were set up using the general linear model, and group data were analyzed in a random-effects model. Statistical parametric maps were generated using *t* statistics to identify regions activated according to the model. The encoding blocks were contrasted with the control blocks. All reported activations passed a threshold of p<0.001 using FDR correction (cluster size k≥10 voxels).

The effect of memory self-efficacy beliefs on encoding was assessed by comparing the [Encoding>Control] maps between the low and high memory self-efficacy beliefs groups. Sex was added as a covariate of no interest. Results were considered significant at p<0.001 uncorrected, k≥10 voxels.

Median-split comparisons were preferred to correlations because one participant, a 20 year-old man with the highest PRMQ score (score = 54), tended to modify the strength of the correlations but not their trajectories. This participant did not show extreme brain effects in the comparative approach. Therefore, the median-split comparison was more conservative and highlighted the most consistent effects related to memory self-efficacy beliefs scores. Nonetheless, the correlational analyses can be found in Figure S1, providing further validation of the findings.

## Results

### Behavioral Results

Table 1 displays scores obtained at the PRMQ and Swahili task on day 1, and the cognitive tasks performed outside the scanner on day 9. As aforementioned, in contrast with Crawford et al. [21] who did not find any effect of sex on the PRMQ, we observed a trend toward a sex effect (see Table 1), such that 5 out of 6 women were categorized in the low memory self-efficacy group, and 7 out of 10 men were categorized in the high memory self-efficacy group. When controlling for this confounding variable, only performance in feature binding significantly differed between the 2 groups, where the low-memory believers performed less well than the high-memory believers. Trends were observed for manipulation of visuospatial information in working memory (low<high memory believers) and for associative learning (low>high memory believers). The latter trend may be due to an immediate, reactive effect of completing the PRMQ. Indeed, since the Swahili associative memory task took place just after completion of the PRMQ, it might be that the low memory self-efficacy believers were more motivated and tried harder in a compensatory attempt.

### Neuroimaging Results

#### Encoding versus control

**Figure 2:**
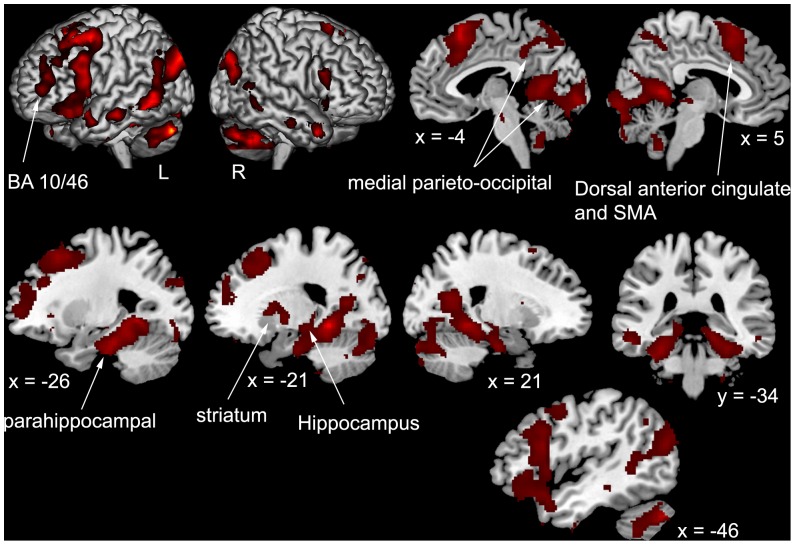
Pattern of brain activations in all subjects when encoding intentions. Overlay of the activation map [Encoding>Control] on a standard MNI brain. BA = Brodmann Area; L = left; R = right, SMA = supplementary motor area.

Encoding future intentions elicited brain activity change in left prefrontal (PFC) areas (ventrolateral, dorsolateral and rostrolateral regions), bilateral parahippocampal cortex (including body and tail of left hippocampus), retrosplenial cortex, occipito-parietal areas (precuneus and calcarine regions), motor and premotor areas as well as the basal ganglia and cerebellum (Table 2, Figure 2). While these activations are typical of episodic encoding, the pattern differs from that found in studies of imagining future scenarios where medial prefrontal areas (medial BA 10), associated with the self, were shown [39]. Even at a very permissive threshold (p<0.05 uncorrected), no significant activity was found in medial PFC during encoding in comparison with the control task.

**Table 2:** Brain activity during the encoding of future intentions in comparison with the control task.

		MNI coordinates				
Regions	BA	x	y	z	t	k
Calcarine, ventral precuneus, retrosplenial, lingual, paraHC, HC (body, tail) LR	27,30,17,18	−14	−62	12	15.82	11294
Cerebellum crus 1 L		−48	−70	−34	12.93	648
Dorsolateral PFC, ventrolateral PFC, dorsomedial PFC, PFC anterior L	8–10, 44–47	−4	22	54	12.85	5636
Occipital and temporal middle, dorsal precuneus L	19–21,37,39,7	−38	−82	34	9.59	3593
Cerebellum lobule 9 LR		−12	−52	−48	9.36	482
Dorsomedial PFC, SMA, Cingulate middle R	6,8,32	4	20	60	9.08	737
Cerebellum crus 2 L		−8	−84	−44	8.70	97
Occipital middle, angular R	19,39	40	−70	30	8.65	722
Temporal pole middle R	38	48	16	−34	8.59	97
Caudate L		−16	10	8	7.33	121
Ventrolateral PFC R	45	56	26	22	7.05	191
Pallidum L		−22	−2	2	6.60	10
Temporal inferior L	20	−42	−4	−46	6.60	29
Temporal inferior R	20,21	54	−6	−28	6.22	90
Temporal middle R	20	58	−38	−12	5.98	118
Caudate R		16	6	14	5.97	54
Dorsolateral PFC R	44	36	12	36	5.90	40
Temporal middle R	21	52	−52	8	5.67	86
PFC anterior R	46	34	54	22	5.24	13
Ventrolateral PFC R	47	54	26	−8	5.24	67

P<0.001 FDR correction, cluster extent k≥10. Voxel size is 2 mm^3^.

Abbreviations: BA = Brodmann Area; HC = hippocampus; L = left; MNI = Montreal Neurological Institute; PFC = prefrontal cortex; R = right; SMA = supplementary motor area.

#### Comparison of encoding-related activity between the low and high memory self-efficacy believers

**Figure 3:**
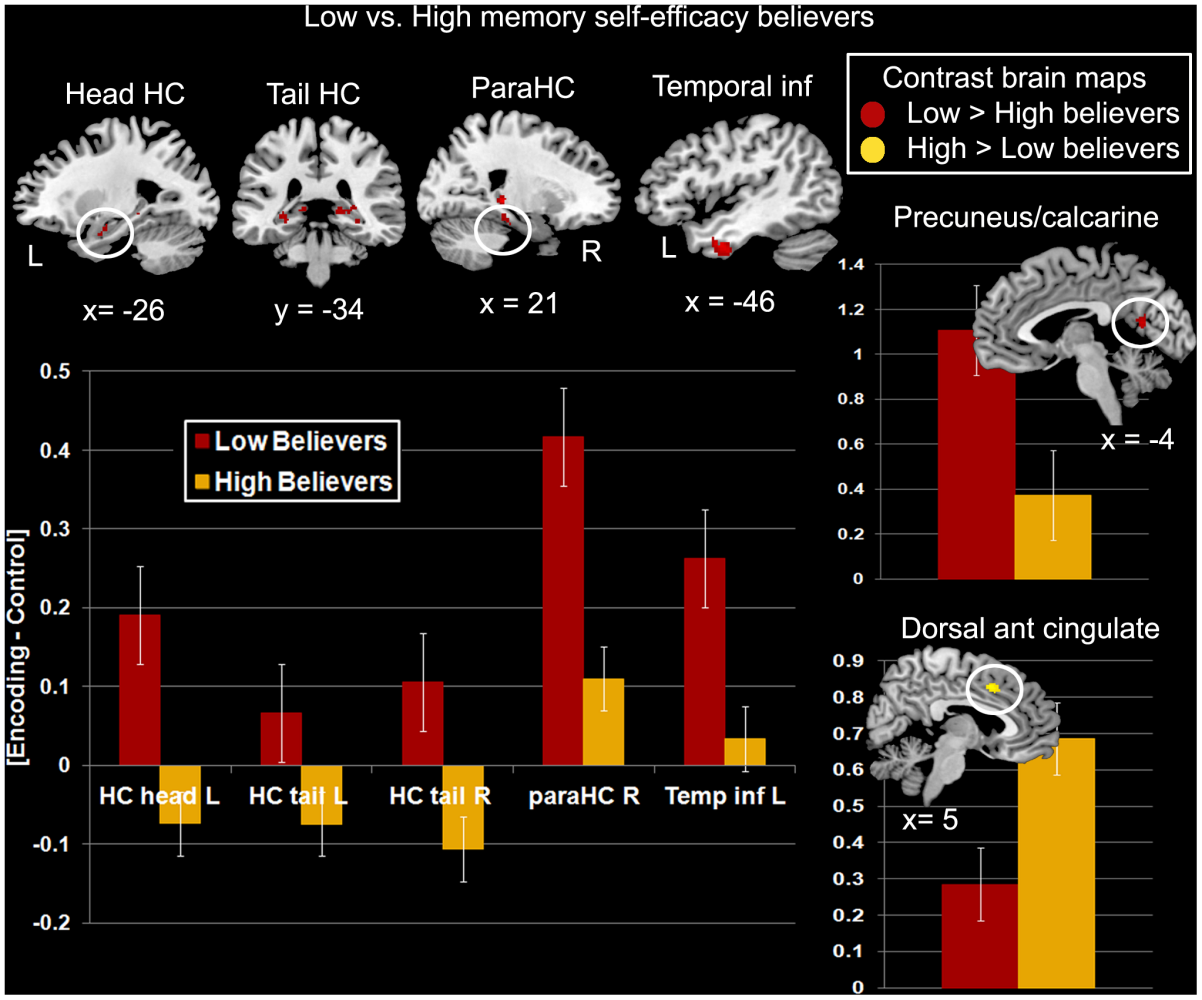
Differences in brain activity during encoding between the low and high memory self-efficacy believers. Group differences in brain activity is overlaid on a standard MNI brain: Red clusters represent regions where more activity was found in the low-memory beliefs group in comparison with the high-memory beliefs group; the yellow cluster denotes the region more activated by the high-memory beliefs group in comparison with the other group. For the sake of comparison with the most engaged regions during encoding in the whole group, the same sagittal and coronal slices are shown in Figure 2. The graphs display the mean difference in activity between the encoding and control conditions in each group (red: low-memory believers; yellow: high-memory believers) for the highlighted regions. Values on y-axes represent beta values from the comparison analyses. Dorsal ant cingulate = dorsal part of the anterior cingulate gyrus; HC = hippocampus; L = left; ParaHC = parahippocampal cortex; R = right; Temporal inf = inferior temporal cortex.

**Figure 4:**
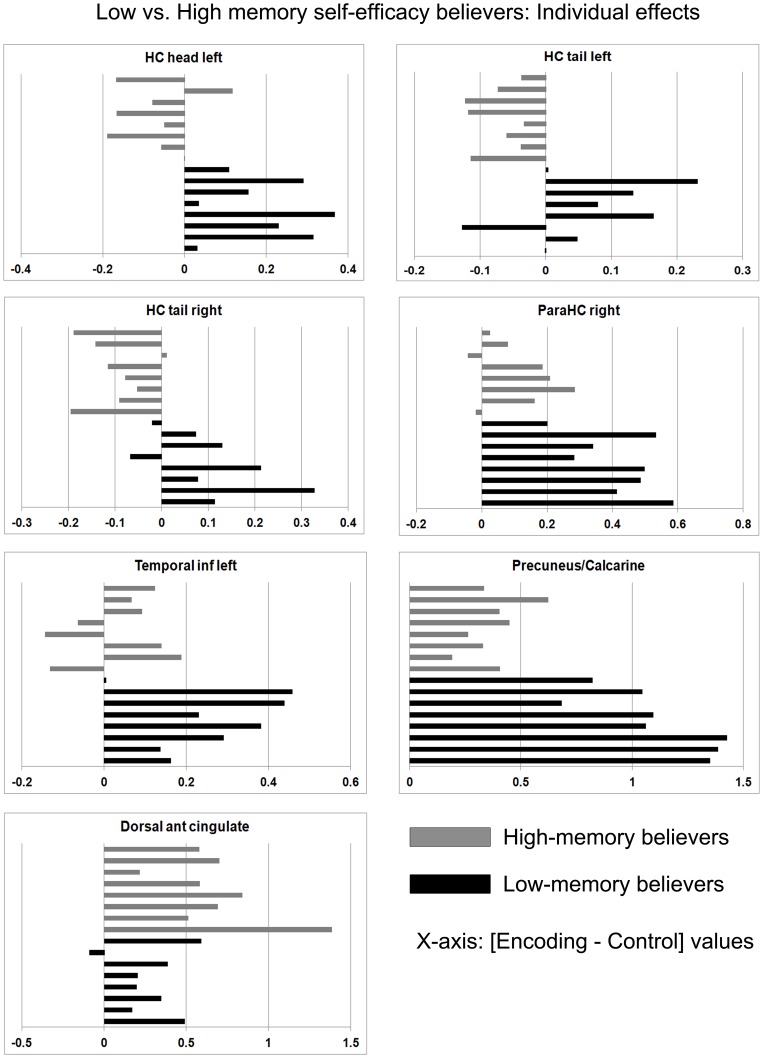
Individual encoding-related brain activity within the low vs. high memory self-efficacy comparison. These graphs show the detail of the group effects in Figure 3 at the individual level.

Controlling for sex, the low-memory beliefs group showed more activity than the high-memory beliefs group in hippocampus (left head and bilateral tail), ventral precuneus and calcarine, right parahippocampal cortex, cerebellum, left inferior temporal cortex, and thalamus. The inverse contrast showed more activity for the high-memory believers in the dorsal part of the anterior cingulate cortex (Table 3, Figure 3). In Figure 3, it was also interesting to note that the hippocampi were activated during encoding only by the low-memory beliefs group, while both groups activated the other regions but with a significant difference in level of activity (ventral precuneus/calcarine, parahippocampal, inferior lateral temporal and dorsal anterior cingulate regions). The impression from the group level results was further strengthened by the individual activity plots shown in Figure 4.

**Table 3:** Brain activity differences when encoding future intentions.

		MNI coordinates				
Regions	BA	x	y	z	t	k
Low>High memory self-efficacy believers						
Hippocampus tail R		18	−30	2	6.18	52
		30	−34	−8	4.06	10
Hippocampus tail L		−30	−36	0	5.76	34
		−14	−32	2	4.78	15
Hippocampus head/amygdala L		−26	−6	−18	4.18	14
Parahippocampus R	30	22	−26	−18	4.52	21
Temporal inferior L	20	−44	−6	−38	5.75	117
Cerebellum lobule 6 R		14	−62	−24	5.43	15
Precuneus/calcarine L	30,23,17	−2	−60	14	5.23	52
Cerebellum crus 1 R		38	−46	−36	5.02	32
Thalamus medial LR		0	−14	0	4.56	26
High>Low memory self-efficacy believers						
Cingulate anterior dorsal R	24	4	12	44	4.56	27

P<0.001 uncorrected, k≥10 voxels, and adjusted for sex.

Abbreviations: BA = Brodmann Area; L = left; MNI = Montreal Neurological Institute; R = right.

## Discussion

The main aim of this study was to investigate the effect of general memory self-efficacy beliefs for everyday life situations as measured with the PRMQ [21] on brain activity when individuals were asked to memorize future real-world intentions, which were later performed in a virtual reality environment [12]. Activity in several brain areas was modulated according to memory self-efficacy beliefs. Differences at separate cognitive tasks were also observed, such that low-memory believers performed less accurately at feature binding and, at trend, manipulating visuospatial information in working memory. After a discussion on the brain areas involved when encoding intentions in the whole sample, we discuss the relationships between memory self-efficacy beliefs, cognitive performances and brain activity.

### Pattern of Brain Areas Engaged when Encoding Intentions

In this experiment, participants were asked to visualize themselves performing real-world tasks at specific locations in the center of their residential town. Knowledge of these locations was examined the day before the experiment, and when visualizing themselves in short scenarios, the participants knew they would have to subsequently perform them in a virtual simulation of the town. Thus, in contrast to previous investigations on prospection, the imagined scenes had to be encoded in memory in order to be acted out in the near future.

We found prefrontal areas typically activated during episodic encoding such as ventrolateral PFC (BA 44, 45, 47), dorsolateral PFC including BA 9/46 (for reviews see [1], [40], [41]) and more dorsal regions like the superior frontal sulcus involved in visuospatial working memory [42], [43], [44]. The pattern of activations was strongly left-lateralized, in line with the HERA model (Hemispheric Encoding/Retrieval Asymmetry, [45]), which predicts left PFC activity during episodic encoding. Left rostrolateral PFC (BA 10/46) was initially found in episodic retrieval rather than encoding tasks (for meta-analyses, see [1], [2]). Whereas Botzung et al. [46] found activity in this area during evocation of both future and past events and concluded that this region may support mental time travel, Addis et al. [47], who directly compared brain activity during the elaboration of past and future events, found this region to be more active for future thinking. Consequently, they proposed that this region could be specialized in prospection (see also [48] for a meta-analysis that corroborates this interpretation). Furthermore, it has been hypothesized that encoding future intentions requires recovery of past experiences. Hence, left rostrolateral PFC would subtend the retrieval of past personal events in order to generate an internal contextual support for intention representation [49]. The rostrolateral BA10 activity found in our study may thus reflect episodic retrieval mechanisms in the service of encoding future actions.

Massive activity was found in medial temporal lobe, especially in the parahippocampal cortex bilaterally. Its involvement in episodic encoding and retrieval has been frequently reported [2], and more particularly for item-based processing [50]. The parahippocampal cortex has also been related to visuospatial mnemonic processes, and it would be more precisely engaged in allocentric (viewer-independent) spatial representations (see [51] for review). Thus, it is possible that when encoding intentions to be performed at specific places, the individuals retrieved in their memory each place and its location, according to their personal knowledge but also to the “place+map” items presented the day before scanning. Activity was found in the body of the left hippocampus (Figure 2), fitting well with a previous finding where activity in the body of the left hippocampus was modulated by the amount of detail provided by the individuals concerning future but no past events [52]. Prince et al. [53] revealed that the body of the left hippocampus was the only region to survive a conjunction analysis involving semantic and perceptual encoding and retrieval. Thus, this subregion would play a crucial role in retrieving and recombining multimodal elements from the past into a new event [47].

Encoding intentions induced activation of medial parietal regions, including the retrosplenial cortex and the precuneus (Figure 2). The function of the retrosplenial cortex in spatial memory has been previously documented, and some authors reported that it would be involved in representing allocentric landmark direction [54]. According to Moscovitch et al.'s model of spatial and episodic memory [54], the precuneus would be implicated in egocentric imagery. Together with the activation of occipito-temporal regions, all the aforementioned regions (hippocampus, parahippocampal cortex, retrosplenial cortex, and precuneus) constitute a network, specialized in the mental representation of locations within an environment, and its integration into episodic memory.

Among the studies where mental imagery of simple or complex scenarios was required, only a few of them highlighted basal ganglia activity [5], [47], [55]. Szameitat et al. [56] suggested that the involvement of basal ganglia when imagining everyday actions (swimming, eating) was related to the storage and retrieval of motor programs. Basal ganglia have also been found in experiments requiring temporal organization of motor sequencing [57]. Thus, in addition to motor and premotor areas [58], basal ganglia (and especially the caudate in the present task) may be important for the encoding of complex actions for later enactment, and could have a function in the temporal organization of motor sequencing [59].

Previous studies on future thinking emphasized the involvement of midline antero-posterior regions, largely recognized as being part of the default mode network. The default mode network is defined as characterizing brain activity in the absence of goals directed toward the external world. Similarities between this network and activations found in tasks that rely on the self (autobiographical remembering, projection into the future, theory of mind) have led to a strong connection between this mental state and internal simulation of scenarios [39], [60]. While the present study showed activity of posterior midline regions, it contrasts with previous studies by the absence of medial prefrontal areas (BA 10/11) [5], [6], [7], [46]. Similarly to Poppenk et al.'s study [10] (see also [58]), we did not find medial BA 10 activation even at more permissive statistical thresholds. Thus, the act of intentionally encoding delayed intentions may not recruit medial BA 10 activity to the same extent as imagination and elaboration of plausible events, without the requirement of encoding, would.

### Modulation of Memory Self-efficacy Beliefs on Encoding

Activity in several brain areas was modulated according to memory self-efficacy beliefs. More activity was found in the low memory self-efficacy believers in comparison with the high-memory believers mainly in medial temporal lobe areas, precuneus/calcarine and left lateral temporal cortex. By contrast, the high-memory believers showed more activity only in the dorsal anterior cingulate cortex. This indicates that when intentionally prompted to memorize new information, different neurocognitive systems and/or different levels of similar cognitive operations engaged during the task may take place according to beliefs in memory self-efficacy. In other words, individuals may apprehend a task differently depending on their beliefs [23].

Those with low memory self-efficacy beliefs also demonstrated reduced performance in feature binding (and, at trend, manipulating visuospatial information in working memory). The hypothetical awareness of weaker cognitive abilities in everyday life might be a global determinant of the variability in memory self-efficacy [14], and may likely constitute the factor that has induced modulation in brain activity. To our knowledge, only one study has explored whether the PRMQ was linked to objective measures of cognitive functions in healthy young adults, other than objective memory measures. Mäntylä et al. [61] examined the relationships between PRMQ scores and executive functions. The rationale behind this study was that executive functions, which are highly required in everyday-life tasks (e.g., for goal-directed behavior control), may impact the perception of self-efficacy beliefs including beliefs about prospective memory functioning. Mäntylä et al. [61] found an association between PRMQ scores and shifting, but not between PRMQ scores and inhibition or updating. Here too, we did not find any difference between the 2 groups in updating and inhibition. To our knowledge, no study has investigated the relationships between PRMQ scores and other cognitive domains such as attention and working memory subcomponents other than the aforementioned executive functions. Complementing Mäntylä et al.’s findings, we here showed that individuals who reported more everyday memory failures also showed more difficulties in specific working memory subcomponents, namely feature binding and, at trend, manipulation of visuospatial information. In Gonneaud et al. [31], feature binding has been shown to be a strong mediator between aging and impairment in event-based prospective memory, which supports the notion that feature binding may be an important cognitive function for successful encoding of future intentions. It has to be noted that we used the same feature binding task as in [31] with a few modifications (e.g., longer retention time in the present study, to avoid ceiling effects, as the subjects were all young adults). Thus, in line with Mäntylä et al.’s hypothesis, difficulties in feature binding and manipulating visuospatial information in working memory may reduce people’s self-confidence in memory capabilities. Hence, memory self-efficacy beliefs may act as a mediator between individual working memory differences and modulation in brain activity when encoding information.

Low-memory believers displayed activity increase in regions of the medial temporal lobe and ventral precuneus/calcarine areas. As aforementioned, these regions have been previously shown to be involved in episodic memory tasks, and especially when the tasks included visuospatial elements. In contrast with the general encoding pattern shown across all participants, particular parts of the hippocampus were more activated by the low-memory believers: posterior hippocampus bilaterally and the head of the left hippocampus. Although functional specialization of the hippocampus on its anterior-posterior axis remains unclear, some relevant structural and functional neuroimaging findings from previous investigations provide some clues. One function of posterior hippocampus may be process-based such that it is more engaged during episodic retrieval rather than encoding [62]. Recently, it has been shown that posterior hippocampus is engaged during the encoding of familiar scenes but not to novelty, and that its activation predicts subsequent memory [63]. Accordingly, low-memory believers may engage more retrieval processes of the previously seen or known locations to improve encoding. The head of the hippocampus, also more activated in the low-memory believers, has been related to binding processes (for review see [64]), but as we noted above, so is the body of the hippocampus. Focusing on the spatial aspect of the task, a recent study showed that when imagining ourselves in a given environment with increasing number of boundaries, activity in the hippocampus was only modulated by vividness, and not actual or perceived difficulty, or complexity of the environment [65]. In line with Addis et al. [52], this supports the hypothesis that left hippocampal activity is modulated by vividness of imagined events. This would suggest that low-memory believers may have visualized themselves at specific locations more vividly than the high-memory believers, reflecting strategic mechanisms based on visuospatial processes to enhance encoding. However, we did not evaluate vividness of the visualized scenes, and this hypothesis requires to be tested in future experiments.

Overall, in line with our hypothesis mentioned in the Introduction and according to which participants with low memory self-efficacy beliefs would either enhance or use different cognitive processes, we suggest that they recruited cognitive processes in which they were generally less efficient (feature binding, manipulating visuospatial information) to a greater extent in order to execute the required task (encoding intentions) efficiently. The instructions further encouraged the subjects to use a visuospatial strategy, which may have contributed to the increased activity in brain areas supporting feature binding and manipulation of visuospatial features like the hippocampus, parahippocampal cortex and medial occipito-parietal regions. The left lateral temporal cortex was also more activated in the low-memory believers group, suggesting additional verbal processes when memorizing the future intentions. This higher engagement may reflect the fact that low-memory believers might have been more motivated to efficiently achieve the encoding task. Studies in which motivation has been manipulated (e.g., memory tasks with reward and/or punishment) showed a higher engagement of the basal ganglia, nuclei in which we did not find signal change. However, some studies showed a significant correlation between activity in striatal nuclei and hippocampus related to reward during successful encoding in episodic memory (e.g., [66]), the hippocampus being connected to the reward circuit. Thus, it is also possible that higher hippocampal activity found in the low-memory beliefs group actually reflects higher motivation to perform well, and possibly engaging or enhancing binding and visuospatial cognitive processes.

The only brain region that was more activated in the high-memory beliefs group was the dorsal part of the anterior cingulate gyrus. This area is part of a larger fronto-cingulo-parietal network subserving executive mechanisms supporting cognitive control, as recently confirmed in a meta-analysis including results of 193 studies [67]. We also recently showed that the anterior cingulate cortex was more activated for successfully retrieved items that were previously repeatedly retrieved and therefore better consolidated [38]. Hence, it is possible that high-memory believers had higher skills in visualizing themselves in locations that already had better mnemonic representation. However, this hypothesis would need to be tested more specifically, in addition to the fact that in the present study the highlighted area was more posterior to that found in Eriksson et al. [38].

Alternatively, the increase of activity in brain structures associated with visuospatial mnemonic processing (i.e., hippocampus and precuneus) seen for low-memory believers, combined with the activity increase in cognitive-control regions (anterior cingulate) for high-memory believers may reflect a strategic difference such that high-memory believers may focus more exclusively on core aspects of each mnemonic event. Thus, low-memory believers may consider a richer scenario during encoding, whereas high-memory believers may consider less features of each event. However, this seems contradictory with the behavioral results, where low-memory believers had poorer performance on feature binding (richness/complexity) rather than inhibition (focus on specific aspects).

An effect that was not initially expected was sex difference in memory self-efficacy beliefs, where more women were categorized as low-memory believers whereas more men were categorized in the high-memory beliefs group. This sex effect fits with cognitive differences that we observed at visuospatial working memory tasks, as women usually perform less well than men [68]. As we were not interested in sex effects in the present study, we controlled for this factor in all comparisons. Moreover, this observation must be considered cautiously as this study enrolled a small number of participants. Potential sex differences in self-efficacy beliefs should be further pursued with larger sample size of genders.

A limitation of this study is that we could not test potential relationships between memory self-efficacy beliefs and prospective memory performance. If previous investigations did not evidence a significant link between PRMQ scores and prospective memory performance, probably due to the nature of the tasks (laboratory tasks, which lacked ecological validity), here we could have expected a significant relationship because the task was naturalistic. This question could not be addressed because of experimentally induced ceiling performance during prospective retrieval. Therefore, it is not known whether the additional brain activity revealed at encoding in the low-memory believers could have had efficiently compensated for their assumed lower capabilities in everyday life. Even though the effect of self-efficacy beliefs on memory performance was not the main aim of the study (but rather their effects on how an intentional encoding memory task is handled), it would be interesting to also address this question, as findings so far have shown a weak relationship between subjective and objective prospective memory tests [30]. Nonetheless, our findings do support the hypothesis that subjective memory is linked to objective cognitive abilities (e.g., feature binding) that are important for prospective memory function. Although we support the view that subjective memory may partly result from objective cognitive abilities, the relationships between these dimensions, including also personality for instance, is likely to be complex. Finally, although we do not have evidence from the participants’ feedback that filling in the PRMQ a day before the scanning session had had any influence on their behavior during the tasks, this possibility cannot be rejected. It may also reflect what happens in everyday life, where it has been shown in naturalistic settings that people with low memory self-efficacy beliefs use strategies to compensate for their prospective memory shortcomings [23]. To disentangle this issue, future studies on larger study samples should vary the order or time-window between the metamemory questionnaire and memory task in order to verify whether such a manipulation leads or not to different results.

## Conclusion

The present study shed the light on the effects of memory self-efficacy beliefs on brain activation during encoding of real-life intentions. Specifically, low-memory believers showed increased activity in brain areas related to visuospatial episodic memory (hippocampus, medial parieto-occipital regions), while high-memory believers showed increased activity in dorsal anterior cingulate cortex, which subserves cognitive control. Based on the observation of cognitive differences in feature binding and manipulation in working memory capabilities between the 2 groups, we concluded that the increased activity seen in the low memory self-efficacy believers may reflect enhancement of these cognitive operations necessary for efficient encoding; this effect may have been linked to motivational and compensatory processes. These results constitute a basis for future research on the effects of subjective memory on neurocognitive mechanisms related to everyday-life behavior.

## Supporting Information

Figure S1 Correlations between PRMQ scores (X-axis) and [Encoding – Control] activity (adjusted beta values at the peaks, Y-axis) in the whole sample.
(PDF)

Text S1 Description of the offline cognitive tasks.
(PDF)
