# Supplementary material for: Memory Self-Efficacy Beliefs Modulate Brain Activity when Encoding Real-World Future Intentions
Source: PLoS One. 2013 Sep 3;8(9):e73850. doi: 10.1371/journal.pone.0073850 (PMC3760799; doi:10.1371/journal.pone.0073850)
Supplement: Figure S1 — Correlations between PRMQ scores (X-axis) and [Encoding – Control] activity (adjusted beta values at the peaks, Y-axis) in the whole sample. (PDF) [file pone.0073850.s001.pdf]

Supporting Information: Results

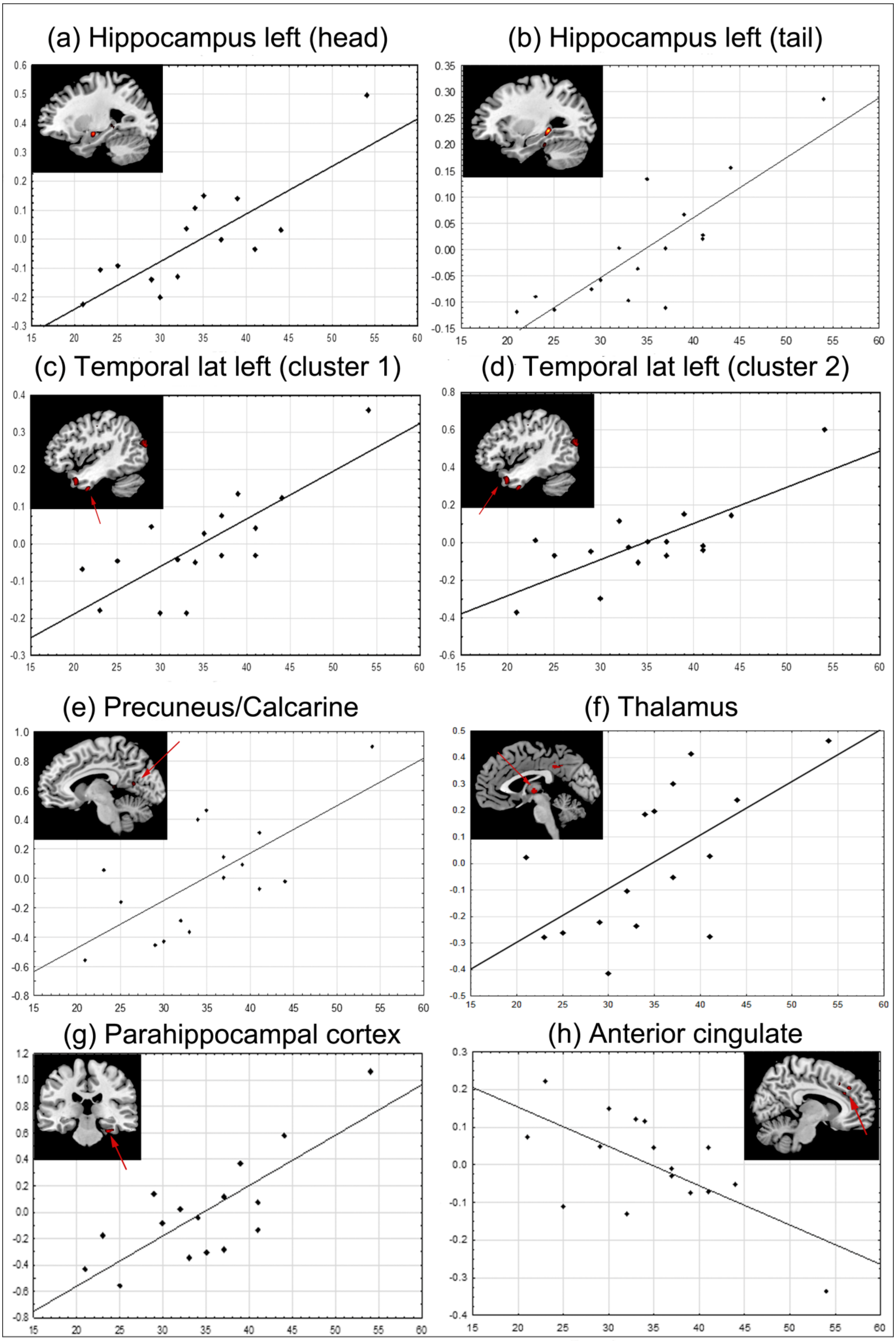

Correlations between PRMQ scores (X-axis) and [Encoding – Control] activity (adjusted beta values at the peaks, Y-axis) in the whole sample. (a) Left hippocampus (head, peak coordinates [-26;-10;-12]) with t-value = 6.11; plot without controlling for sex ($r = 0.79$, $p < 0.001$; r after controlling for sex = 0.86, p one-tailed < 0.001). (b) Tail of the left hippocampus [-30;-36;-4] $t = 7.88$; plot without controlling for sex ($r = 0.83$, $p < 0.001$; r after controlling for sex = 0.91, $p < 0.001$). In the correlation analysis, the effect in the right hippocampal tail was smaller ($r = 0.58$, $p = 0.01$; r after controlling for sex = 0.63, $p = 0.005$). (c, d) There were 2 clusters in the left lateral temporal cortex. (c) [-46;-4;-40] $t = 5.59$; (d) [-44;14;-30] $t = 5.39$; plot without controlling for sex for (c) ($r = 0.77$, $p < 0.001$; r after controlling for sex = 0.84, $p < 0.001$), and (d) $r = 0.76$, $p < 0.001$ (r after controlling for sex = 0.83, $p < 0.001$). (e) Calcarine/precuneus [-8;-60;12] $t = 4.18$; plot without controlling for sex ($r = 0.70$, $p = 0.002$; r after controlling for sex = 0.76, $p < 0.001$). (f) Thalamus [-2;-12;2] $t = 3.29$; plot without controlling for sex ($r = 0.62$, $p = 0.005$; r after controlling for sex = 0.67, $p = 0.003$). (g) In the correlational analysis, there was a cluster close to the right parahippocampal cortex, but located more inferior to the cluster of the median-split analysis, in the fusiform and cerebellum. Plot for the right fusiform cortex [30;-30;-26] (close to the parahippocampal cortex) without controlling for sex ($r = 0.78$, $p < 0.001$; r after controlling for sex = 0.85, $p < 0.001$). (h) Dorsal anterior cingulate cortex [12;22;42] $t = 3.65$; plot without controlling for sex ($r = -0.65$, $p = 0.003$; r after controlling for sex = -0.71, $p = 0.002$).

In addition, a positive correlation between PRMQ and activity was found in the left occipital cortex [-42;-84;24] $t = 6.59$, $k = 192$; plot without controlling for sex ($r = 0.81$, $p < 0.001$; r after controlling for sex = 0.88, $p < 0.001$). This correlation was driven by the outlier, however when computing the correlation without him, $r = 0.66$, $p = 0.004$ (and $r = 0.67$, $p = 0.005$ after controlling for sex). In the median-split analysis, this cluster appeared at $p = 0.004$, $t = 3.16$, $k = 11$, therefore it did not pass the threshold we set ($p < 0.001$).

Correlations between PRMQ and cognitive performance: Only the correlation between PRMQ and binding was significant (r after controlling for sex = -0.57, p two-tailed = 0.03), replicating the behavioral results based on median-split presented in the manuscript.
