# Supplementary material for: Memory Self-Efficacy Beliefs Modulate Brain Activity when Encoding Real-World Future Intentions
Source: PLoS One. 2013 Sep 3;8(9):e73850. doi: 10.1371/journal.pone.0073850 (PMC3760799; doi:10.1371/journal.pone.0073850)
Supplement: Text S1 — Description of the offline cognitive tasks. (PDF) [file pone.0073850.s002.pdf]

Supporting information Text S1: Methods

Feature binding: The binding task measured the ability to associate visuospatial features in working memory. Four colored uppercase letters were displayed in the center of a 5x4 grid, accompanied with 4 colored crosses displayed randomly in the other squares of the grid. The task was to mentally associate each of the 4 colored letters with the location of the cross of the same color. Three seconds were allocated to this encoding phase. After a black fixation cross appearing at the center of the blank screen for 8 sec, a new grid appeared with only one black lowercase letter in one of the squares. The task was to decide as fast as possible if the letter was in the correct square (press the D key if correct, L key if incorrect). The next trial started with a 2-sec fixation cross when a response has been provided. In total, 40 trials were administered.

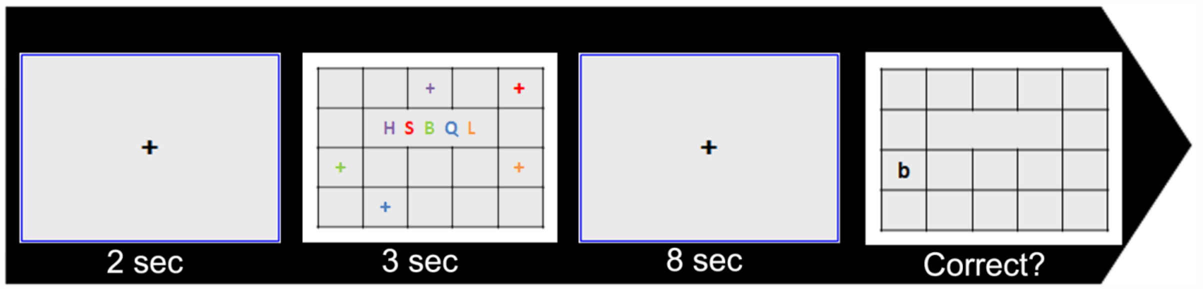

Maintenance and manipulation of visuospatial information in working memory: A computerized version of the Corsi cubes was used, forward (for maintenance) and backward (for manipulation). In the forward task, a series of keys to be reproduced in the same order on the keyboard of the computer appeared on the screen. The minimum number of consecutive keys was 4 (and maximum 11). Three trials for each length were assessed, even if one (or more) was successful. In the backward task, the minimum length was 3 keys (and maximum 11), and 3 trials of each length were assessed. Both tasks were finished when the participant failed at the 3 trials of a given length. Each key was highlighted for 1 sec. Instead of using span as score, we used the number of correct trials to increase between-subject variability.

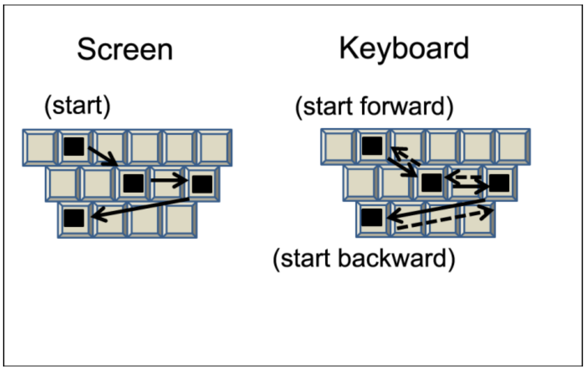

Exogenous (bottom-up) and exogenous (top-down) attention: The Posner paradigm was assessed as follows: 252 intermixed trials composed the exogenous attention task: 144 valid (72 left, 72 right), 36 invalid, 48 neutral and 24 null trials. The instructions were to always keep the eyes focused on the central cross. The left or right highlighted square signaled the cue; participants were informed that the subsequent target had 80% chance to fall in the same position as the cue (valid trials), and 20% chance to fall in the opposite square (invalid trials). If both the left and right squares were highlighted, there was an equal chance that the target fell in either the left or right square. No target appearance constituted the null trials. The instructions were to press D (left) or L (right) as accurately and fast as possible to indicate target's position. A trial was displayed as follows: 1) fixation: 2 s, 2) cue: 100 ms, 3) fixation: 200 ms, 4) target: 50 ms, 5) fixation response: 2 s. The endogenous attention task included 252 intermixed trials; the same number of valid, invalid, neutral and null trials as in the exogenous task were used. An arrow positioned in the central square constituted the cue, indicating either the left or right squares (or both for the neutral trials). A trial was displayed as follows: 1) fixation: 2 s, 2) cue: 100 ms, 3) fixation: 50 ms, 4) target: 50 ms, 5) fixation response: 2 s. The score was the difference of reaction time between the invalid and valid trials, indicating speed of attention re-orientation.

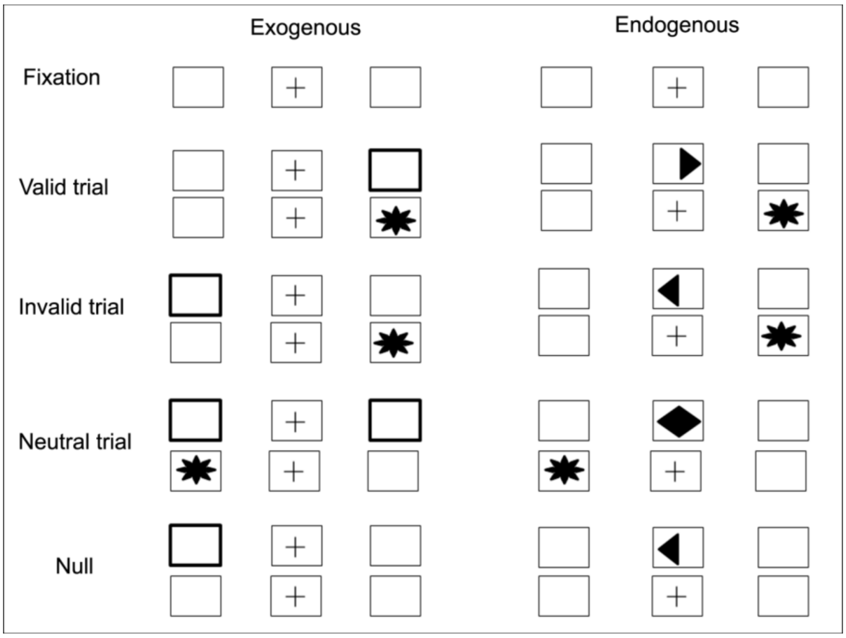

Updating: The letter memory task was used to measure updating efficiency. Ten series of upper-case letters (A, B, C, D) of variable length (from 7 to 15 letters) were displayed one by one on the screen, for one second each. Subjects were not informed of the length of each series. The task was to remember in the correct order the 4 last letters. The score was the number of correct responses.
